# Reply to M Mialon et al.

**DOI:** 10.1016/j.advnut.2023.07.002

**Published:** 2023-09-13

**Authors:** Vivica I. Kraak

**Affiliations:** From the Department of Human Nutrition, Foods, and Exercise, Virginia Polytechnic Institute and State University (Virginia Tech), 257 Wallace Hall, 295 West Campus Drive, Blacksburg, VA 24061

Dear Editor:

I was heartened that my perspective paper concerning conflicts of interest (COIs) for professional service within the 2020 Dietary Guidelines Advisory Committee (DGAC) [1] was read by Mialon et al. [2], who published on this topic in 2022. Managing COIs and biases is complex to navigate. In my perspective paper, a key point raised to address these authors’ spurious methods was that “defining and describing the COI context accurately is essential given the variation across institutions and professional activities” [1]. I clearly defined the differences between individual and institutional COIs and biases, which differ based on whether one is conducting research or serving as an expert advisory committee in the United States [1].

The data availability statement of the paper by Mialon et al. [2] indicated that “the raw data used in this manuscript was derived from publicly available resources.” Their letter stated that “It is not clear to us how information already made public by DGAC members themselves could bring any harm to their reputations.” However, these researchers datamined the publicly available curriculum vitae of 20 United States researchers, removed the information from the original context, published their names (personally identifiable information) in a peer-reviewed journal article without the consent of the DGAC members, and failed to provide sufficient evidence to support their findings.

These authors should reflect on whether they have upheld data ethics standards for conducting research and complied with United States data privacy protection laws. Several of these authors may be familiar with the European Union’s and United Kingdom’s General Data Protection Regulation (GDPR), which is guided by the following 7 principles: lawfulness, fairness, and transparency; purpose limitation; data minimization; accuracy; storage limitations; integrity and confidentiality; and accountability [3].

Three distinct policy bodies have different reporting time frames for COI disclosures ranging from 1 y to 4 y. No authoritative body requires COI reporting by professionals over 20 y who are either engaged in grant-funded research or appointed to an advisory committee. The United States Office of Research Integrity (ORI) policy and confidential form requires each DGAC member to declare relevant COIs over 12 mo or 1 y [4]. The International Committee of Medical Journal Editors provides a form used by many medical and public health journals that require the disclosure of COIs for the previous 36 mo or 3 y [5]. The COI policy of the World Health Organization (WHO) for advisory committee service is 48 mo or 4 y. The WHO policy applies to “current interests that have arisen during the four years preceding the WHO work, and COI does not apply to past interests that have expired or that no longer exist, nor does it apply to possible interests that may arise in the future but which do not currently exist” [6]. Mialon et al. [2] used an unreasonable standard to make public judgments about the eligibility of DGAC members for professional service.

I am unaware that any of these authors have served on or staffed an expert advisory committee. My professional experience of staffing expert committees at the National Academies (2002–2007) has taught me that a competing interest, whether financial or nonfinancial, for an individual member will have very limited influence on how a consensus committee evaluates the evidence to develop findings. When convening expert advisory committees, United States government agencies may follow different due diligence processes, although most strive to balance diverse expertise and cognitive biases. Mialon et al. [2] have remained silent on how the collective COIs or biases of the 20 DGAC members associated with the food and pharmaceutical industries had influenced the findings or recommendations of the 835-page 2020 DGAC scientific report [11].

I believe that there is a greater risk of COIs and biases influencing the translation of the DGAC scientific report into the 2020–2025 Dietary Guidelines for Americans (DGA) report[12]. However, the process used by the Secretaries of the United States Department of Health and Human Services (HHS) and Agriculture (USDA) during the Trump Administration, and a comparison of the content of the DGAC and DGA documents, remain unexamined. The HHS and USDA staff reported recent progress to strengthen transparency for the 2025–2030 DGA process [7]. However, these agencies could report the individual COI disclosures for the 2025 DGAC members rather than a summary of the collective COIs, as requested by 14 advocacy organizations [8] to increase transparency and public trust in their future work.

I agree with the authors [2] that documenting COIs and biases for professionals is essential to build a culture of trust in science and democracy, especially in an age of misinformation [9]. According to McNutt and Crow [9], “Science is slow and focuses on generating knowledge to improve decision-making, whereas misinformation scrambles the meaning of knowledge and its ability to further the public good…To advance the public good, scientists must also assess the moral bases of their pursuits, both in conducting research and communicating the results to the public.” Mialon et al. [2] could have demonstrated better professional judgment to use their study findings for more constructive ends. The readers of this journal, who are your academic peers, will judge the merits of your study and how you communicated the findings to the public.

As a scholar employed at a United States academic institution, I am required by my university and the HHS ORI to complete an online Collaborative Institutional Training Initiative (CITI) course every 4 y [10]. The course refreshes my understanding and compliance with the ORI’s 4 types of conflicts described in my article, and I take full responsibility to be held accountable for what needs to be disclosed, how it needs to be disclosed, and to whom, with permissible activities. The CITI course is useful for researchers at all phases of their professional careers to ensure ethical practices when conducting rigorous research and to comply with legal requirements [10].

## Author contribution

The author’s responsibilities were as follows – The sole author was responsible for all aspects of this manuscript.

## Conflict of interest

The content is solely the responsibility of the author and does not necessarily represent the official views of the USDA. This research did not involve human subjects; therefore, it was exempt from institutional review board requirements. VIK has not served or staffed a federal U.S. government Dietary Guidelines Advisory Committee. VIK served on the NASEM Food Forum (2016-2022) and is an ASN member. VIK did not receive any funding from the commercial or private-sector entities for research or consulting, and has no financial or non-financial COIs related to the content of this manuscript.

## Funding

This manuscript was supported by the Virginia Tech’s Department of Human Nutrition, Foods, and Exercise; and the United States Department of Agriculture (USDA), National Institute of Food and Agriculture, Hatch project VA-160189.
